# Increased intracellular proteolysis reduces disease severity in an ER stress–associated dwarfism

**DOI:** 10.1172/JCI93094

**Published:** 2017-09-18

**Authors:** Lorna A. Mullan, Ewa J. Mularczyk, Louise H. Kung, Mitra Forouhan, Jordan M.A. Wragg, Royston Goodacre, John F. Bateman, Eileithyia Swanton, Michael D. Briggs, Raymond P. Boot-Handford

**Affiliations:** 1 Wellcome Trust Centre for Cell-Matrix Research,; 2 Faculty of Biology, Medicine and Health, and Manchester Academic Health Science Centre, Manchester, United Kingdom.; 3 Murdoch Children’s Research Institute, Parkville, Victoria, Australia.; 4 School of Chemistry and Manchester Institute of Biotechnology, Faculty of Science and Engineering, University of Manchester, Manchester, United Kingdom.; 5 Institute of Genetic Medicine, Newcastle University, Newcastle upon Tyne, United Kingdom.

**Keywords:** Bone Biology, Development, Cartilage, Collagens, Genetic diseases

## Abstract

The short-limbed dwarfism metaphyseal chondrodysplasia type Schmid (MCDS) is linked to mutations in type X collagen, which increase ER stress by inducing misfolding of the mutant protein and subsequently disrupting hypertrophic chondrocyte differentiation. Here, we show that carbamazepine (CBZ), an autophagy-stimulating drug that is clinically approved for the treatment of seizures and bipolar disease, reduced the ER stress induced by 4 different MCDS-causing mutant forms of collagen X in human cell culture. Depending on the nature of the mutation, CBZ application stimulated proteolysis of misfolded collagen X by either autophagy or proteasomal degradation, thereby reducing intracellular accumulation of mutant collagen. In MCDS mice expressing the *Col10a1.*pN617K mutation, CBZ reduced the MCDS-associated expansion of the growth plate hypertrophic zone, attenuated enhanced expression of ER stress markers such as *Bip* and *Atf4*, increased bone growth, and reduced skeletal dysplasia. CBZ produced these beneficial effects by reducing the MCDS-associated abnormalities in hypertrophic chondrocyte differentiation. Stimulation of intracellular proteolysis using CBZ treatment may therefore be a clinically viable way of treating the ER stress–associated dwarfism MCDS.

## Introduction

Mutations in collagen X cause the short-limbed dwarfism metaphyseal chondrodysplasia type Schmid (MCDS) (1–3). Mutant collagen X proteins misfold and are retained within the ER of hypertrophic chondrocytes (4), causing an increase in ER stress and activation of the unfolded protein response (UPR), ultimately disrupting growth-plate hypertrophic cell differentiation and bone growth (5, 6). Indeed, triggering increased ER stress by targeting the expression of an exogenous misfolding protein (*cog* form of thyroglobulin) to hypertrophic chondrocytes is sufficient to induce an MCDS-like phenotype, demonstrating the central role of ER stress in the disease mechanism (6). The presence of increased ER stress in hypertrophic chondrocytes causes the cells to disengage their differentiation program in an attempt to reduce ER stress and survive (5, 6). The altered differentiation program causes a decreased production of VEGF by hypertrophic chondrocytes, resulting in delayed vascular invasion and a characteristic expansion in the hypertrophic zone width (5, 6).

Carbamazepine (CBZ) is an FDA-approved drug for use in epilepsy, bipolar disorder, and neuropathic pain (7). CBZ also stimulates both autophagy and proteasomal degradation pathways and thereby has been shown to prevent liver fibrosis occurring as a result of misfolded protein accumulation in a mouse model of α1-antitrypsin deficiency (8–10). Here, we show that CBZ is able to reduce ER stress caused by the intracellular accumulation of MCDS-causing forms of collagen X by stimulating their intracellular degradation through autophagy or proteasomal degradation, depending on the particular mutation. In vivo in an MCDS mouse model, which expresses the MCDS-causing N617K mutation in collagen X, CBZ induced a reduction in ER stress and enabled hypertrophic chondrocytes to improve their differentiation, resulting in increased bone growth rates and reduced skeletal dysplasia.

## Results and Discussion

**Figure 1:**
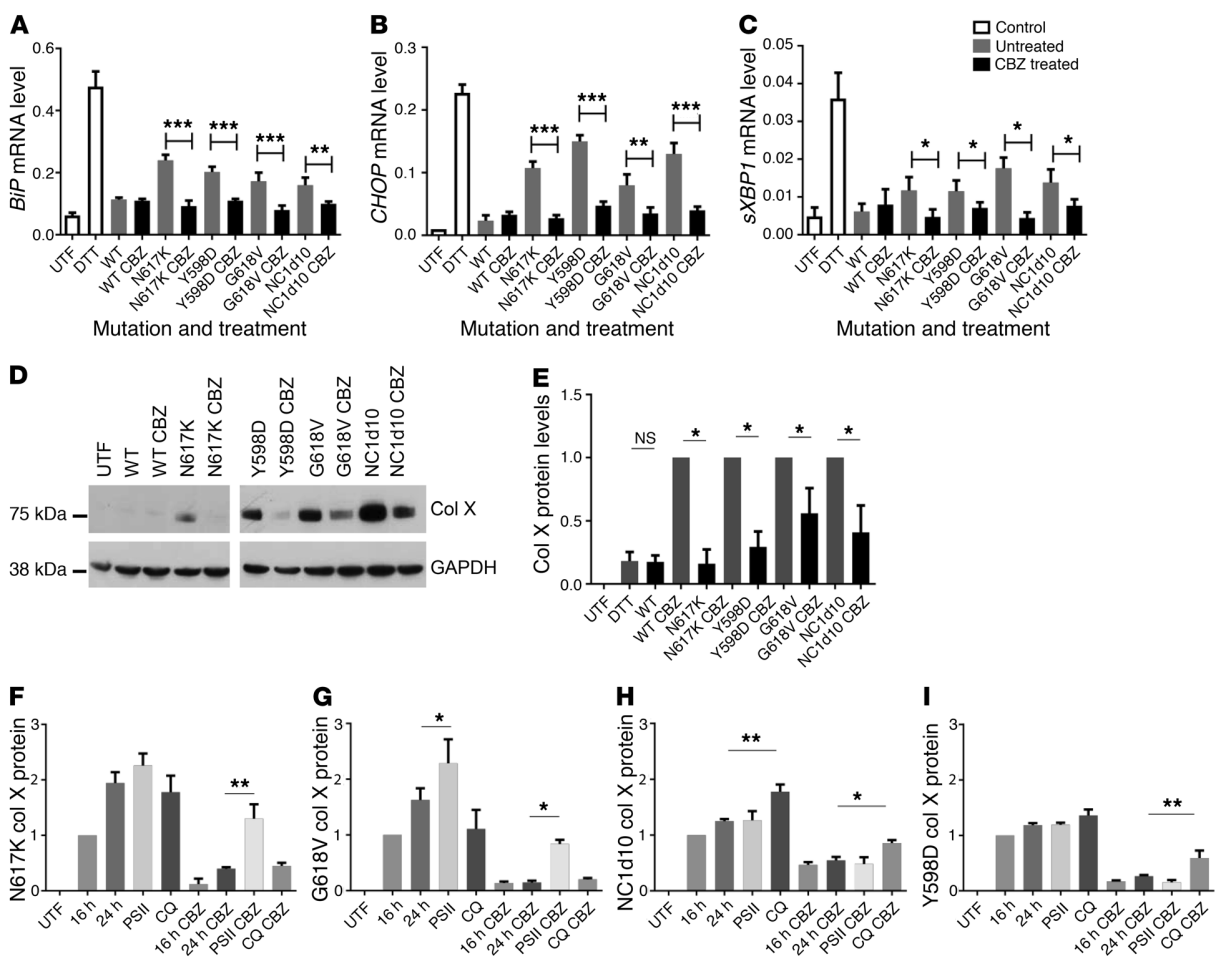
Effects of CBZ treatment on ER stress induced by 4 different MCDS-causing mutations in collagen X. (**A**) *BiP*, (**B**) *CHOP*, and (**C**) spliced *XBP1* (*sXBP1*) mRNA (relative to β actin mRNA) in cells transiently expressing 1 of 4 mutant collagen X constructs and treated for 24 hours with CBZ (20 μM). Mean ± SEM (*n* = 4). UTF, untransfected control. (**D**) Western blot of intracellular collagen X (Col X) protein levels with and without 24-hour CBZ treatment and (**E**) quantification of 3 independent experiments (mean ± SEM). (**F**–**I**) Quantification of 3 independent Western blot experiments for intracellular N617K, G618V, Nc1del10, and Y598D collagen X protein levels (75 kD) in the presence or absence of 20 μM CBZ for 24 hours (see Supplemental Figure 2D for representative blots). Inhibitors of the proteasome (PSII) or lysosome (CQ) were added at 16 hours after transfection for a further 8 hours. Collagen is expressed relative to its GAPDH level and was normalized to the retained collagen X level at the 16-hour untreated time point. Mean ± SEM. *P < 0.05; **P < 0.005; ***P < 0.0005. All statistical analyses by ANOVA.

We hypothesized that drugs capable of reducing the ER stress caused by mutant forms of collagen X may be of use as potential treatments for MCDS. We therefore screened our MCDS cell culture model (HeLa cells transiently expressing the MCDS-causing N617K collagen X mutation) with a panel of compounds that act as chemical chaperones and/or relieve ER stress, including sodium phenylbutyrate (11), CBZ (10), tauroursodeoxycholic acid (11), glycerol (12), dimethyl suphoxide (13), verapamil (14), and quercetin (15). CBZ was the only compound tested that reduced the levels for each of the ER stress–induced mRNAs, *BiP*, *CHOP*, and spliced *XBP1* (Supplemental Figure 1; supplemental material available online with this article; https://doi.org/10.1172/JCI93094DS1). Furthermore, CBZ was able to significantly reduce the mRNA levels for these genes in cells expressing 4 different MCDS-causing collagen X mutations (N617K, Y598D, G618V, and NC1del10; ref. 4) (Figure 1, A–C). For each of these mutations, the CBZ-induced reduction in ER stress was accompanied by significant reductions in the levels of intracellularly retained mutant collagen X protein (Figure 1, D and E). Next, we focused on the rates of degradation to explain the CBZ-induced reduction in mutant protein accumulation, since the drug did not affect cell viability, collagen X mRNA levels, or rates of general protein synthesis (Supplemental Figure 2, A–C). CBZ is a known stimulator of autophagy via mTOR-independent mechanisms (8), but has also been shown to stimulate degradation via proteasomal mechanisms (9). We tested the effects of both proteasomal (proteasome inhibitor II [PSII]) and autophagy (chloroquine [CQ]) inhibitors on the accumulation of mutant collagen X in the cell culture system in the presence and absence of CBZ. In the absence of CBZ, mutant collagen X accumulated over a 24-hour period (Figure 1, F–I, and Supplemental Figure 2D). For N617K and G618V, intracellular accumulation was enhanced by the proteasomal — but not by the autophagy — inhibitor, which was added for the final 8 hours of culture (Figure 1, F and G, and Supplemental Figure 2D). In contrast, the intracellular accumulation of NC1del10 and Y598D was enhanced by the autophagy — but not the proteasomal — inhibitor (Figure 1, H and I, and Supplemental Figure 2D). CBZ treatment reduced the intracellular accumulation of each mutant protein without altering its degradation pathway (Figure 1, F–I, and Supplemental Figure 2D) or its secretion into the medium (data not shown). CBZ was therefore able to stimulate the degradation of mutant collagen X by both autophagy and ER-associated degradation via the proteasome. The different pathways by which N617K/G618V compared with NC1del10/Y598D mutations were degraded presumably relate to their abilities to be resolved from a misfolded aggregate to a single polypeptide chain, with the latter being degraded via the proteasome and the aggregated forms of collagen by autophagy as described previously (16).

**Figure 2:**
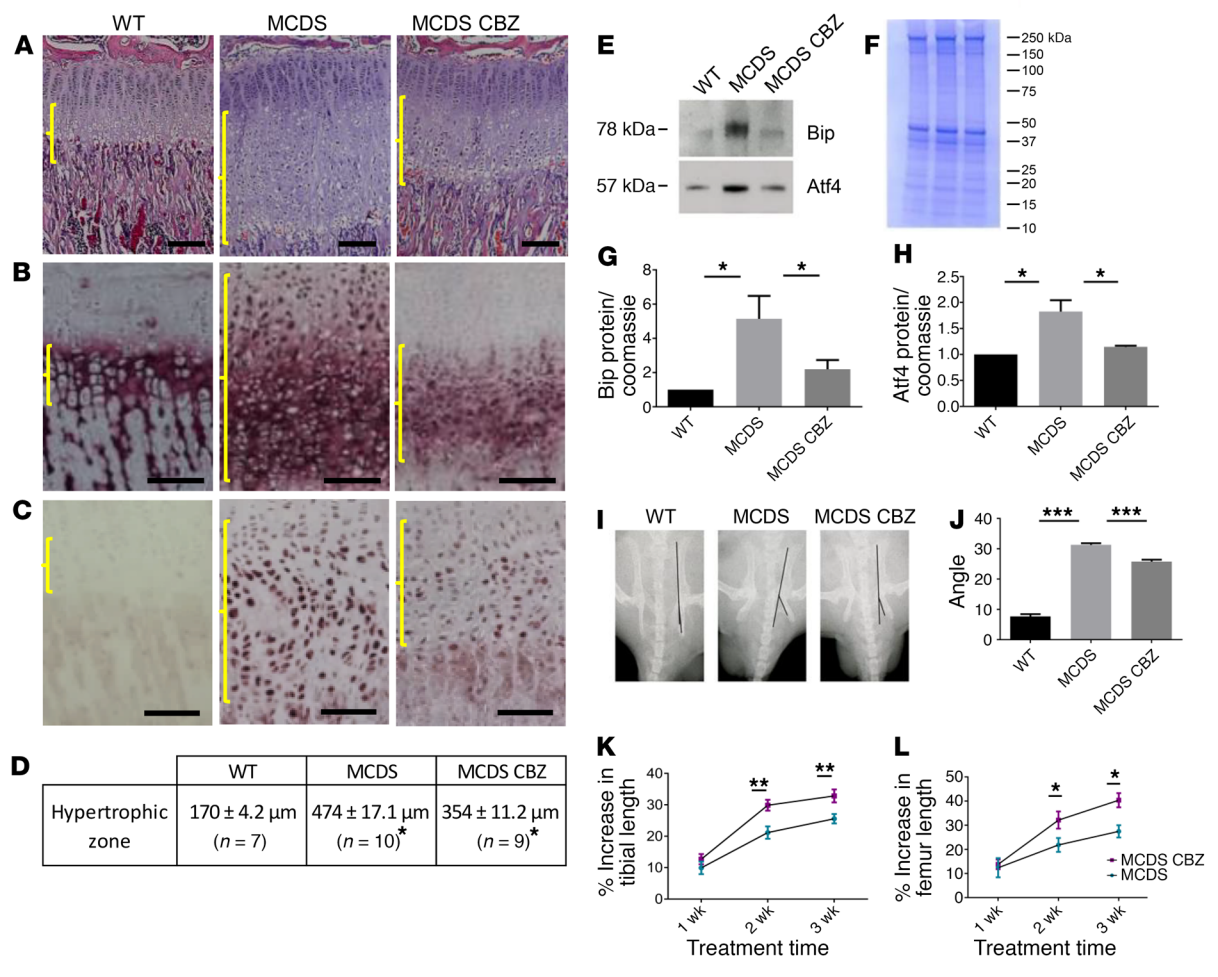
Effect of CBZ on the growth plate pathology associated with MCDS. Three-week-old MCDS mice were treated with CBZ for a period of 1 week. Untreated MCDS mice and mice WT for collagen X were used as controls. (**A**) H&E staining of the tibial growth plate and immunohistochemistry for (**B**) collagen X and (**C**) Bip/Grp78. Brackets indicate the hypertrophic zone. Scale bars: 100 μM. (**D**) Hypertrophic zone measurements at 4 weeks of age. Mean ± SEM. MCDS vs MCDS CBZ *P < 0.05. (**E**) Typical Western blots of rib growth plate cartilage extracts at 4 weeks of age for Bip and Atf4 alongside their Coomassie blue–stained protein loading control gel (**F**). (**G** and **H**) Quantification of BiP and Atf4 (*n* = 3 independent analyses). (**I**) X-ray images of pelvis illustrating the distortion of the ischial tuberosity in MCDS mice at 6 weeks of age. (**J**) The angle between the lines was measured for each group (mean ± SEM, *n* = 5). Bone growth expressed as percentage of increase based on length at 3 weeks of age in each animal for (**K**) tibia and (**L**) femur. Mean ± SEM. *n* = 6 MCDS mice and *n* = 7 MCDS CBZ-treated mice. *P < 0.05; **P < 0.005; ***P < 0.0005. All statistical analyses by ANOVA.

In order to examine the effects of CBZ in vivo, we treated MCDS mice with CBZ (250 mg/kg body weight/d) for 1 to 3 weeks. This dose level of CBZ in mice is equivalent to that routinely used in children to treat epilepsy and related disorders when species differences in drug dosage levels are taken into account (17). Mice dosed by gavage achieved a serum CBZ concentration of 4.4 μg/ml 2 hours after ingestion. Newborn MCDS pups treated from E10 by gavaging the pregnant dams displayed a 40% reduction in the expansion of the growth plate width seen in the untreated MCDS pups (*P* = 0.0001; Supplemental Figure 3). Untreated 3-week-old MCDS mice displayed the characteristic expansion in the width of the hypertrophic zone compared with WT mice. One week of CBZ treatment was sufficient to cause a significant reduction in the width of the hypertrophic zone compared with that in untreated MCDS controls (Figure 2, A and D), and this effect continued throughout the 3-week treatment period (Supplemental Figure 4). These data show that CBZ treatment is capable of reducing MCDS pathology, since it is already established that the degree of hypertrophic zone expansion positively correlates with disease severity (5, 6). CBZ treatment of WT mice had no effect on hypertrophic zone width (Supplemental Figure 5, A and B).

Immunohistochemical analysis of tibial growth plates revealed, as expected, that WT collagen X was secreted into the extracellular matrix surrounding the hypertrophic chondrocyte, whereas in MCDS mice, the mutant protein was retained intracellularly with delayed and reduced collagen X secretion in the lower half of the hypertrophic zone (Figure 2B) (6). The CBZ-induced degradation of misfolded protein described above accounts for the reduced level of intracellular collagen X with no overt increase in secreted collagen X observed in the CBZ-treated MCDS mice (Figure 2C and Supplemental Figure 4). CBZ had no effect on collagen X protein localization in WT mice (Supplemental Figure 5C). MCDS mice displayed a significant increase in immunostaining for Bip (Figure 2C) and in the protein levels of both Bip and Atf4 in growth-plate extracts (Figure 2E), clearly indicating elevated ER stress. CBZ treatment reduced Bip immunostaining in growth plates of MCDS mice (Figure 2C) and significantly reduced the levels of both Bip and Atf4 on Western blots (Figure 2, E–H). Therefore, CBZ treatment appears to reduce ER stress in the growth plate of MCDS mice by directly stimulating the proteolysis of mutant collagen X accumulating in the hypertrophic chondrocytes of MCDS mice.

One feature of the MCDS pathology in mice is the presence of hip dysplasia — characterized by an increase in the angle of deflection of the ischial tuberosity (6). Six-week-old WT mice had an average deflection of 7.7° compared with 31.6° in the untreated MCDS mice (Figure 2, I and J). CBZ treatment for 3 weeks reduced this deflection to 25.4°, illustrating that relatively short periods of CBZ treatment have the capacity to improve MCDS-associated skeletal dysplasia (Figure 2, I and J). In addition, 3 weeks of CBZ treatment resulted in significant increases in the rates of long bone growth (Figure 2, K and L). Three weeks of CBZ treatment promoted 1.25- and 1.44-fold increases in the lengths of tibial and femur bone growth, respectively, in MCDS mice (Figure 2, H and I; mean ± SEM [% increase in bone length over treatment period], tibia: untreated 25.6 ± 1.3 [*n* = 6] vs. CBZ 32.7 ± 1.6 [*n* = 7] *P* < 0.05; femur: untreated 27.8 ± 2.3 [*n* = 6] vs. CBZ 40.1 ± 2.0 [*n* = 7], *P* < 0.05). CBZ treatment did not cause a significant increase in bone growth rates in WT mice (Supplemental Figure 4, D and E).

**Figure 3:**
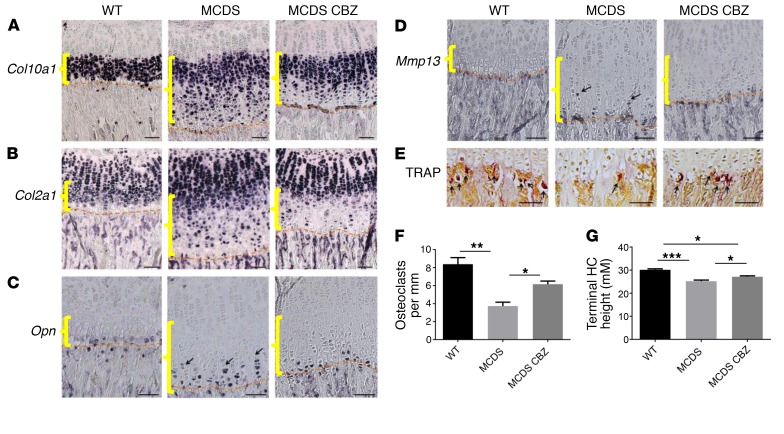
Effect of CBZ on chondrocyte differentiation. MCDS mice were treated with CBZ for 1 week. In situ hybridization for (**A**) collagen X (*Col10a1*) mRNA, (**B**) collagen II (*Col2a1*) mRNA, (**C**) *Opn* mRNA (arrows), and (**D**) *Mmp13* mRNA (arrows). (**E**) TRAP staining for osteoclasts (arrows). (**F**) Number of osteoclasts per mm of vascular invasion front. Mean ± SEM (*n* = 4). (**G**) Final height attained by hypertrophic chondrocytes adjacent to vascular invasion front. Scale bars: 100 μM. Mean ± SEM (*n* = 5)**.** *P < 0.05; **P < 0.005; ***P < 0.0005. All statistical analyses by ANOVA. Yellow brackets show hypertrophic zone; dashed lines show vascular invasion front.

The altered differentiation of hypertrophic chondrocytes caused by increased ER stress in MCDS (5, 6) is characterized by a disrupted pattern of collagen X gene expression, particularly in the lower half of the hypertrophic zone (Figure 3A), accompanied by a sporadic reexpression of collagen II mRNA (Figure 3B). CBZ treatment of MCDS mice reduced the disruption of collagen X and collagen II mRNA expression in the hypertrophic zone (Figure 3, A and B). In WT mice, the *Opn* and *Mmp13* mRNAs are expressed in a coordinated fashion by the terminal hypertrophic chondrocytes immediately adjacent to the vascular invasion front, whereas in MCDS mice, coordination of differentiation is lost and cells autonomously express these terminal markers in the lower half of the expanded hypertrophic zone (Figure 3, C and D) (5, 6). CBZ treatment of MCDS mice caused *Opn* gene expression to become more focused at or close to the vascular invasion front (Figure 3C) and made *Mmp13* gene expression indistinguishable from that of the WT controls (Figure 3D). In addition, CBZ treatment of MCDS mice not only partially corrected the reduced osteoclast recruitment to the vascular invasion front (Figure 3, E and F), but also improved cell hypertrophy, based on the height of the terminal hypertrophic chondrocytes (Figure 3G) (5, 6). These data clearly indicate that CBZ treatment improves the disrupted hypertrophic differentiation apparent in MCDS mice. The lack of effect in WT mice shows CBZ does not have obvious effects on normal physiological processes, as is also suggested by its long and successful clinical use for the treatment of other unrelated disorders.

In summary, CBZ treatment stimulates the degradation of intracellularly retained MCDS-causing mutant forms of collagen X by either autophagy or proteasomal pathways, depending upon the nature of the particular mutation, reducing the level of ER stress both in vitro and in vivo. The CBZ-induced reduction in ER stress causes less disruption to the differentiation process in hypertrophic chondrocytes, leading to decreased skeletal dysplasia and increased bone growth rates. These data provide evidence for the CBZ-induced stimulation of intracellular proteolysis being a potential treatment strategy for reducing the clinical severity of MCDS, and based on the data presented herein, the drug has recently been licensed by the European Medicines Agency for this purpose (http://www.ema.europa.eu/ema/index.jsp?curl = pages/medicines/human/orphans/2016/11/human_orphan_001864.jsp&mid = WC0b01ac058001d12b). CBZ has the potential to act as a therapeutic agent for a broad range of human connective tissue diseases that involve ER stress resulting from the intracellular accumulation of a mutant structural protein.

## Methods

### Statistics.

Multiple comparisons were performed by 1-way ANOVA using GraphPad Prism 6.0 software. A *P* value of less than 0.05 was considered significant.

### Study approval.

The animal experiments performed in this study were approved by the University of Manchester Animal Welfare and Ethical Review Body. Animals were maintained, handled, and sacrificed in strict accordance with United Kingdom Home Office regulations.

See Supplemental Methods for additional information.

## Author contributions

ES, JFB, MDB, and RPBH designed the study. LAM, EJM, LHK, MF, and JMAW performed the experiments. LAM, EJM, LHK, MF, JFB, ES, JMAW, RG, MDB, and RPBH wrote the manuscript.

## Supplementary Material

Supplemental data

## Version 1 09/14/2017

Electronic publication

## Version 2 09/20/2017

Print issue publication
