# Supplementary material for: Increased intracellular proteolysis reduces disease severity in an ER stress–associated dwarfism
Source: J Clin Invest. 2017 Sep 18;127(10):3861–5. doi: 10.1172/JCI93094 (PMC5617653; doi:10.1172/JCI93094)
Supplement: Supplemental data [file jci-127-93094-s001.pdf]

Supplemental Figures (S1-5) and Material and Methods

Supplemental Figure 1

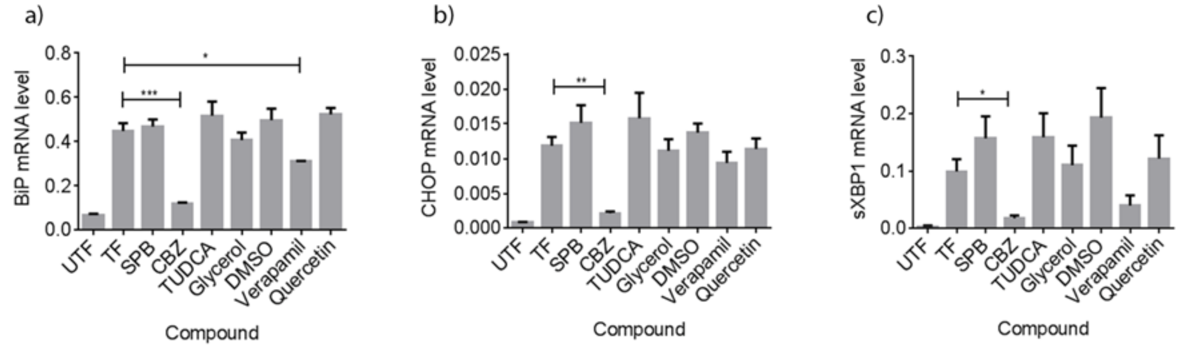

<u>Supplemental Figure 1. Screen for compounds capable of reducing ER stress in vitro</u>

HeLa cells were transiently transfected with N617K collagen X and treated for 24 hours with the compounds indicated. UTF= untransfected control cells, TF= transfected control cells, SPB= sodium phenylbutyrate (1.0 mM). CBZ= carbamazepine (20 µM), TUDCA= tauroursodeoxycholic acid (100 µM), glycerol (0.5% v/v), DMSO= dimyethyl sulfoxide (50 µM), verapamil (50 µM), quercetin (0.1 µM). RNA was extracted from cells and used for qPCR analysis. (a) *BIP* mRNA levels, (b) *CHOP* mRNA levels and (c) spliced *XBP1* mRNA levels relative to β actin mRNA levels. Mean ± SEM (3). *p<0.05, **p<0.005,***p<0.0005

Supplemental Figure 2

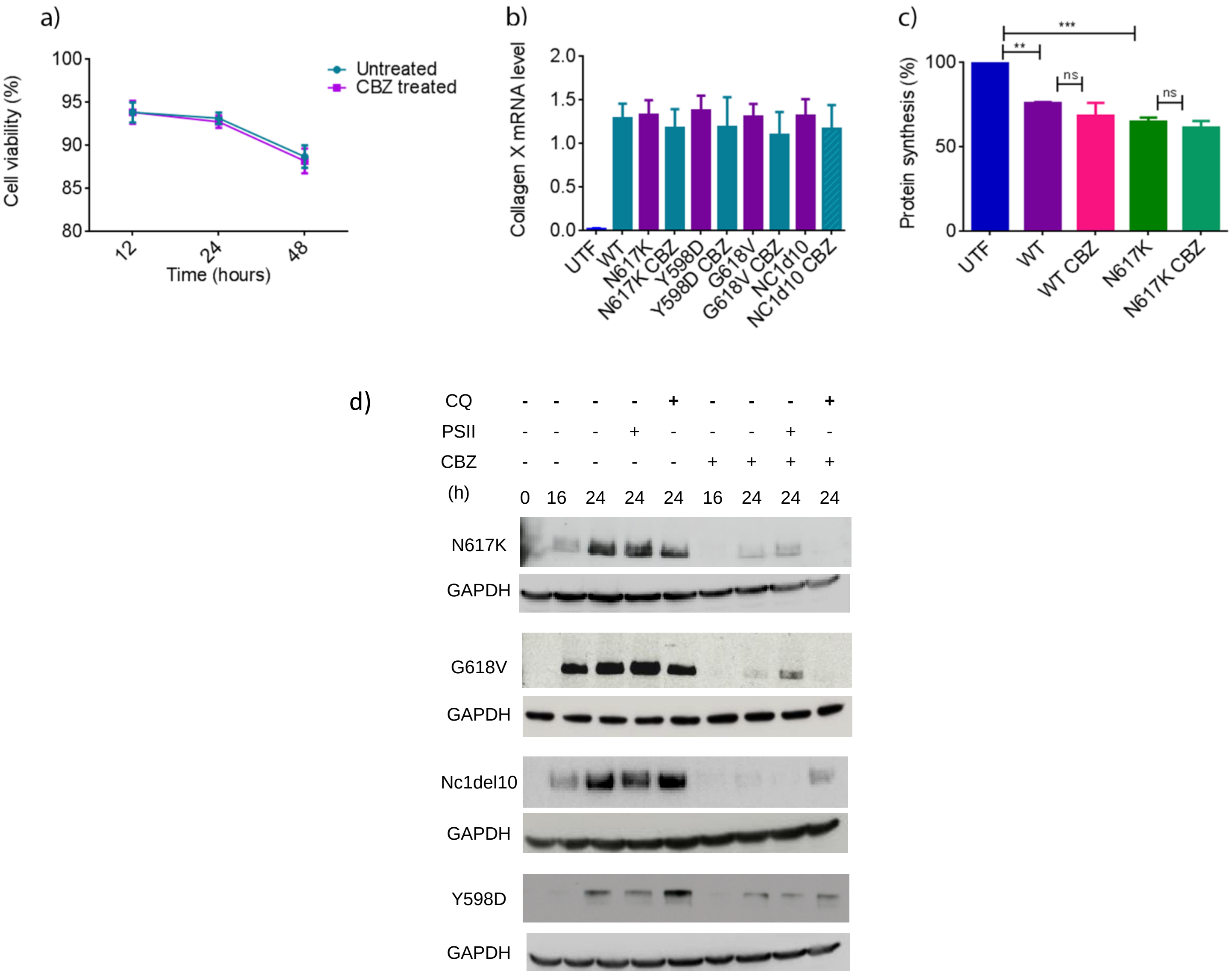

Supplemental Figure 2. Effect of CBZ treatment in vitro

(a) Cell viability of cells treated with 20 µM CBZ for the time indicated. (b) Collagen X mRNA levels in HeLa cells transiently transfected with a range of collagen X mutations in the presence or absence of CBZ for 24 hours. (c) General protein synthesis rates of cells expressing wild-type (WT) or N617K collagen X and treated with CBZ for 24 hours normalised to the untransfected cell (UTF) control. Mean ± SEM (3). $^{**}p<0.005$,$^{***}p<0.0005$, ns = not significant. (d) Representative western blots for the accumulation of intracellular N617K, G618V, Nc1del10 & Y598D collagen X protein levels (75 kD) and their respective GAPDH controls (38 kD) in the presence or absence of 20 µM CBZ for 24 hours (h). Inhibitors of the proteasome (PSII) or lysosome (Chloroquine CQ) were added at 16 h post transfection for a further 8 h. (Quantitation of 3 such experiments is shown in Figure 2f-i).

a)

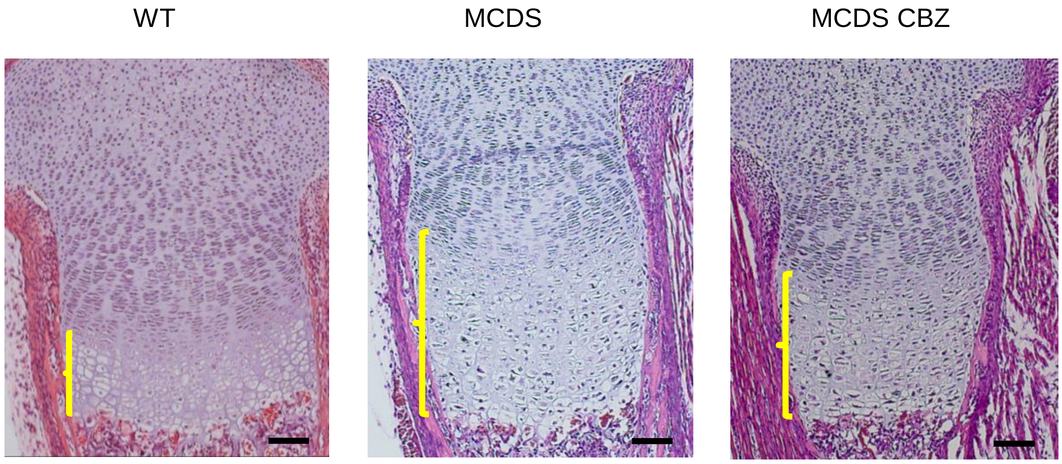

b)

	WT	MCDS	MCDS CBZ
Proliferative zone	383 µm ± 8.5 µm	394 µm ± 15.6 µm	379 µm ± 14.2 µm
Hypertrophic zone	263 µm ± 4.2 µm	399 µm ± 9.0 µm *	345 µm ± 6.2 µm *
	N=5	N=8	N=10

Supplemental Figure 3

Supplemental Figure 3. Effect of pre-natal CBZ treatment on the tibial growth plate pathology in newborn MCDS mice.

Pregnant females carrying offspring homozygous for the p.N617K collagen X mutation were gavaged daily with a 250 mg/kg/day dose of carbamazepine from 10 days post-conception until birth (MCDS CBZ). Pups from untreated pregnant females (MCDS) and females wild-type for collagen X (WT) were used as controls. Pups were sacrificed on the day of birth and tibia analysed for histological purposes. (a) H+E staining of newborn tibial growth plates, hypertrophic zone is marked by the yellow bar. Scale bar equal to 100 µm. (b) Mean measurements ± SEM of hypertrophic zone (HZ) and proliferative zone (PZ) sizes is shown in the table. N= number of animals. *p=0.0001; unpaired t-test

Supplemental Figure 4

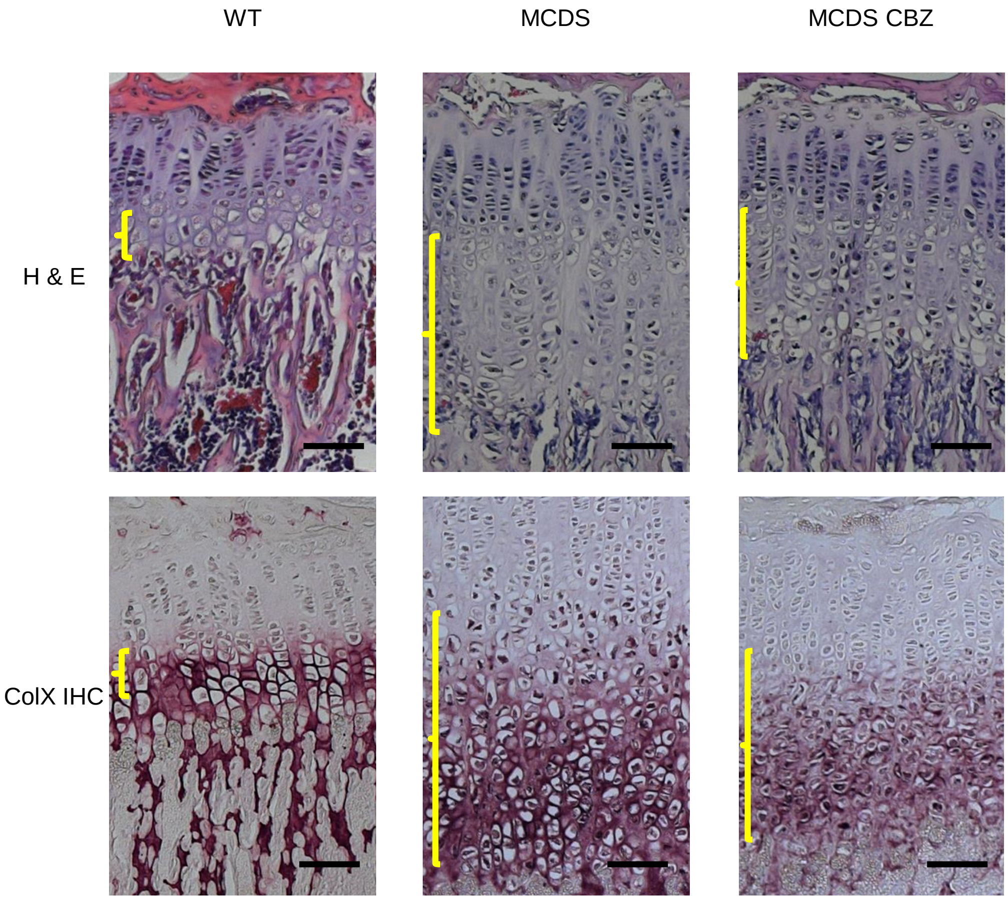

Supplemental Figure 4. Tibial growth plates of 6 week old mice after 3 weeks of CBZ treatment

H & E and Collagen X immunohistochemistry of wild type (WT), MCDS untreated and MCDS mice treated for 3 weeks with CBX (MCDS CBZ). Yellow Bracket = hypertrophic zone; Scale bar = 100 µm.

Supplemental Figure 5

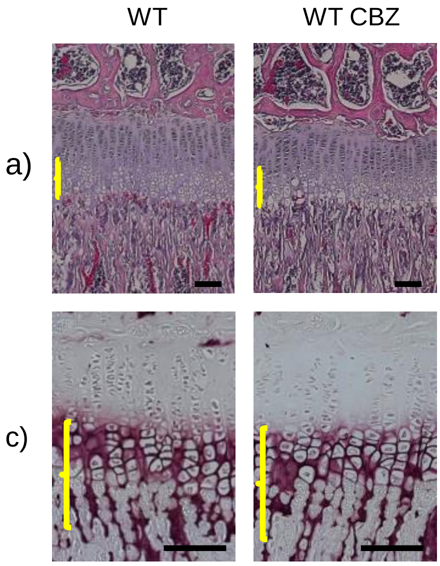

b)

	WT	WT CBZ
Hypertrophic zone	170 ± 4.2 µm (5)	165 ± 7.2 µm (6)

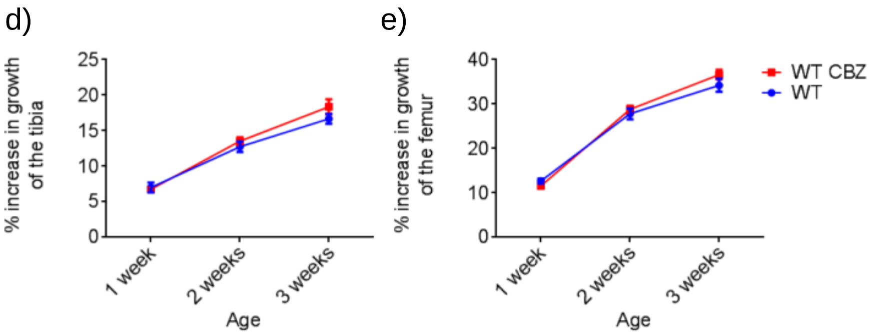

Supplemental Figure 5. Effect of CBZ treatment on wild type mice

Wild-type mice were treated with CBZ (WT CBZ) for a period of one week. Untreated mice (WT) were used as controls. (a) H+E images of the tibial growth plate at 4 weeks of age (yellow brackets = hypertrophic zone; scale bar = 100 µm). (b) Hypertrophic zone widths at 4 weeks of age. Mean ± SEM (number of animals). (c) Immunohistochemistry for collagen X in the tibial growth plate (yellow brackets = hypertrophic zone; scale bar = 100 µm). Bone growth for tibia (d) and femur (e) expressed as a % increase based on length at 3 weeks of age in each animal. Mean ± SEM (n = 7 WT mice and 5 WT CBZ treated mice).

Materials and methods

Antibodies

Antibodies used in this study were as follows: BiP (Santa-Cruz sc-1051), ATF4 (Cell Signalling 11815), LC3B (Sigma L7543), P62 (Santa-Cruz sc-28359), Anti-his (R+D systems MAB050). Collagen X (made in Ray-Boot Handford's lab, University of Manchester).

cDNA Expression Constructs

Human collagen X cDNA pCEP4-WT -His, pCEP4-N617K-His, pCEP4-Y598D-His, pCEP4-G618V-His and pCEP4-NC1del10-His constructs have been described previously (S1).

In vivo experiments

The generation and genotyping of the *Col10a1* p.N617K mouse line has been described previously (S2). Mice homozygous for the MCDS-causing N617K collagen X mutation were x-rayed at 3 weeks of age and treated with carbamazepine (250 mg/kg/day) for up to 21 days via a subcutaneous implantation of a slow-release pellet (Innovative Research of America USA C-113) whilst the mouse was under anaesthetic. The incision was sutured closed, glued using Vetbond tissue adhesive (Santa-Cruz sc-361931) and the mice given an appropriate dose of buprenorphine painkiller depending on their body weight. For some experiments, mice were gavage dosed daily with 0.1 ml/10g BW of 25 mg/ml CBZ dissolved in 75% PEG400/25%dH_2O (v/v). Mice were sacrificed either by cervical dislocation or by carbon dioxide overdose under the provisions of the Animals (Scientific Procedures) Act 1986 and tissues collected for histological analysis. All procedures were carried out according to Home Office regulations.

CBZ serum levels

CBZ serum analysis was conducted on a Q Exactive Plus mass spectrometer connected to a Thermo Ultimate 3000 rapid separation HPLC system that utilised an Accucore C_{18} 100 x 2.1 mm 2.6 µm column for analyte separation (all Thermo Fisher Scientific). A 14 minute targeted mass spectrometry method was developed for ions of a specific mass to charge ratio at a given retention time for carbamazepine in serum. The peak area of the 194 m/z fragment with a retention time of 7.21 minutes (corresponding to CBZ after the loss of the amide side group) was used to quantitate levels.

Skeletal measurements

Mice were anaesthetised using isofluorane, placed on X-Ray hyperfilm (GE healthcare, GZ28906850) and X-Rayed using a Flaxitron X-ray specimen radiography system (Flaxitron MX-20). X-Rays were performed prior to implantation of CBZ pellets and thereafter, once per week for a maximum of 3 weeks. The analysis of the skeleton was done using Growbase software (Certus Technology Associated Limited, UK) to calculate the length of the femur, tibia and inner canthal distance. The bone growth rate was determined as a percentage increase relative to measurements at 3 weeks of age. Image J was used to analyse the angle of deflection from the vertical tuberosity of the ischium to determine the severity of the hip dysplasia phenotype. All measurements were analysed by ANOVA to determine significance using GraphPad Prism 6.0 software.

Histology

Hind limbs were dissected and fixed overnight in ice-cold 4 % paraformaldehyde (w/v) in 1 x DEPC-PBS or 95 % ethanol/5 % acetic acid (v/v), and decalcified in 0.8 M ethyl-diamine tetracetic acid (EDTA) pH 7.4 for a period of one week. The samples were embedded in paraffin wax and sectioned sagittally using a cool-cut HM 355 S microtime (MicRom) generating 5 µm thick sections. Sections

were collected on positively charged superfrost slides (VWR) and dried overnight prior to histological staining, immunohistochemistry or in situ hybridisation.

H+E staining

H+E staining was performed as described previously (S2) and all images taken using a Carl Zeiss Axiovision microscope fitted with an Axiocam colour CCD camera and associated Axiovision software.

Growth plate zone widths were measured on images of known magnification as described previously (S2). The beginning of the proliferative zone was defined as the point at which the individual round chondrocytes flattened out and arranged into columns. The start of the hypertrophic zone was defined as the point at which proliferative chondrocytes rounded up and enlarged. The end of the hypertrophic zone was defined as the vascular invasion front. For each animal the data from three separate sections, spaced a minimum of 50 µm apart were averaged. Measurements were analysed for statistical significance by ANOVA using GraphPad Prism 6.0 software.

Immunohistochemistry

Immunohistochemistry for collagen X and Bip was performed on 95% ethanol/5% acetic acid fixed tibial growth plate sections as described previously (S3).

In situ hybridisation (ISH)

DIG-labelled colourimetric ISH was performed as described previously (S2). The resulting cDNA probes were cloned into a pT7T3 vector then linearized and transcribed using the appropriate restriction enzyme and RNA polymerase.

Tartrate-Resistant Acid Phosphatase (TRAP) staining

Osteoclasts were stained using a TRAP staining kit (Sigma-Aldrich 387A) according to manufacturer's instructions. The number of stained osteoclasts per mm of vascular invasion front was quantified in

3 sections per animal spaced 50 µm apart in 3 animals per genotype and analysed by one-way ANOVA for statistical significance.

Tissue preparation for use in western blotting

Rib-cages from 4 week old mice were dissected and placed in collagenase medium for 1-2 hours, after which the cartilage growth plates were collected using a dissecting microscope. Rib growth plates were then homogenized using a mikro-dismembranator in 100 ul of 2x sample buffer and protein extracted by boiling samples at 99 °C for 10 min and centrifuging at 13,000 rpm for 15 min at 4 °C. The supernatant was collected and analysed by SDS-PAGE.

Cell culture

HeLa cells were cultured at 37 °C with 5 % CO_2 in Dulbecco's modified Eagle's medium (Life Technologies) containing 10 % fetal bovine serum, penicillin (0.5 U/ml)/ streptomycin (0.5 µg/ml), 2 mM L-glutamine, and 1% non-essential amino acids.

Cells were seeded at an appropriate density so that they were approximately 70 % confluent on the day of transfection. The medium surrounding the cells was replaced with fresh culture medium supplemented with 50 µg/ml ascorbic acid and the cells transiently transfected with the collagen X constructs using Lipofectamine 2000. Transfected cells were treated with 20 µM CBZ, 30 min after transfection, for a period of 24 h. Some cultures were also treated with 1 mM DTT for 24 h as a positive control for ER stress

For assessment of the route of protein degradation, transfected cells with or without CBZ treatment were incubated with either a proteasome inhibitor PSII (10 µM) (Calbiochem, 539162) or a lysosomal inhibitor, chloroquine (50 µM) (Sigma, C6628) for the final 8 h of the 24 h culture period and the cell layers then extracted for western blot analyses..

Viability was assessed on trypsinised cells by trypan blue exclusion using a haemocytometer.

Measurement of protein synthesis rate

Cells (in a 12 well plate) were washed twice with PBS before being labelled with 1 ml of media containing 11 µCi EasyTag™ EXPRESS [^{35}S] Protein Labelling Mix (Perkin Elmer NEG772007MC) and incubated at 37 °C for 10 minutes. Cells were rinsed twice with PBS and protein extracted in 100 µl of 2 x SDS-PAGE buffer- 10 µl of which was run on an SDS-PAGE gel. The gel was stained with coomassie blue to determine protein loading before being washed in destain solution for several hours. The gel was fixed in 50% Methanol / 10% Glacial acetic acid, 40% dH_2O (v/v) for 20 minutes and dried in a vacuum gel dryer (BIO-RAD 165-1746) for 2 hours at 85 °C. The dried gel was imaged using a phosphoimager (FLA 3000) and the incorporated counts per sample determined from the resulting image.

Protein extraction in vitro

Protein was extracted from cells 24 h post-transfection. Briefly media was removed and cells washed with 1 X PBS. 100 µl of 2 x SDS PAGE buffer was added per well of a 6 well plate and cells scraped into Eppendorf tubes. Samples were heated to 99 °C for 10 min and centrifuged at 13,000 rpm for 15 min at 4 °C. The resulting supernatant was transferred to a fresh tube and protein concentration determined using a Pierce bicinchoninic acid (BCA) protein assay.

SDS-PAGE and western blotting

20 µg protein per sample was loaded into SDS-PAGE gels. Proteins were separated by SDS-polyacrylamide gel electrophoresis and transferred onto Hybond-P PVDF membrane (LC3) or nitrocellulose membrane (all other proteins). Membranes were blocked using 2% milk powder in PBS containing 0.1% tween-20 for 1 hour at room temperature. The membranes were incubated in primary antibody in blocking solution overnight at 4 ^{o}C at a dilution according to manufacturer's guidelines. The membranes were washed three times in PBS-T and incubated with an appropriate HRP conjugated secondary antibody in blocking buffer for 1 h at room-temperature. Membranes

were washed three times in PBST and developed using an ECL detection kit (Life Technologies) and ECL hyperfilm (GE healthcare).

The blots were quantified by densitometry analysis using ImageJ software, normalised to a loading control and standardised against a control sample on each blot. The results were analysed by ANOVA for statistical significance using GraphPad Prism 6.0 software.

RNA extraction

Media was discarded from wells and the cell layers washed with 1 x PBS. 1 ml of TRIzol reagent (Life Technologies) was added per well of a 6 well plate and incubated for 5 min at room temperature. Cells were scraped into a 1.5 ml Eppendorf tube, 0.2 ml chloroform added and the tube mixed well. Samples were incubated for 3 min at room temperature and then centrifuged at 4 ^{o}C at 10,000 rpm for 20 min. The upper phase was transferred to a fresh Eppendorf and 0.5 ml isopropanol added in order to precipitate the RNA. Samples were mixed well and incubated for 10 min at room temperature before being centrifuged at 10,000 rpm for 15 min at 4 ^{o}C. The supernatant was carefully removed without disturbing the pellet. The pellet was washed in 70 % ethanol and resuspended in 30 µl DEPC- H_20.

RT-qPCR analysis

RNA samples were DNase-treated using the DNA free kit (Applied Biosystems, AM1906) according to manufacturer's instructions in order to remove genomic DNA. 1 µg of RNA was used for reverse transcription using the Taqman Reverse Transcription Reagent Kit (Life technologies UK, 8080234) according to manufactures instructions.

Real time qPCR was performed using the following primers: Collagen X forward 5'CTTCTTTCTCCTTTGCCTG3' and reverse 5'GCTCTCCTCCTTACTGCTATAC3' , *BIP* forward 5' GCTAATGCTTATGGCCTGGA3' and reverse 5'CGCTGGTCAAAGTCTTCTCC3', *CHOP* forward 5' GCGCATGAAGGAGAAAGAAC3' and reverse 5'TCTGGGAAAGGTGGGTAGTG3', spliced *XBP1* forward 5'

GAAGCCAAGGGGAATGAAGT3’ and reverse 5’ CCAGAATGCCCAACAGGATA3’. and *β-actin* forward 5’ CCACCATGTACCCAGGCATT3’ and reverse 5’CACATCTGCTGGAAGGTGGA3’. All reactions were performed in duplicate using a SYBR green kit on an ABIPrismTM 7000 sequence detector system (Applied Biosystems). Each experiment was performed three times in order to confirm the results. Significance determined by ANOVA using GraphPad Prism 6.0 software.

To determine the mRNA level of the spliced form of *XBP1* the cDNA sample was subject to an initial 4 PCR cycles of 95 °C for 3 min, 4 cycles of 95 °C for 40 sec, 60 °C for 45 sec and 72 °C for 40 sec, followed by 72 °C for 10 min in order to create double stranded cDNA. The double stranded cDNA was digested overnight at 37 ^{o}C using a Pst1 restriction enzyme in order to remove the unspliced form of *XBP1*. The resulting digested cDNA sample was then used in RT-qPCR as described above.

Supplemental References

S1. Wilson R, Freddi S, Chan D, Cheah KS, Bateman JF. Misfolding of collagen X chains harboring Schmid metaphyseal chondrodysplasia mutations results in aberrant disulfide bond formation, intracellular retention, and activation of the unfolded protein response. *J Biol Chem.* 2005;280(16):15544-15552.

S2. Rajpar MH*,* et al. Targeted Induction of Endoplasmic Reticulum Stress Induces Cartilage Pathology. *PLoS Genet.* 2009;5(10):e1000691.

S3. Kung LH, Rajpar MH, Preziosi R, Briggs MD, Boot-Handford RP. Increased Classical Endoplasmic Reticulum Stress Is Sufficient to Reduce Chondrocyte Proliferation Rate in the Growth Plate and Decrease Bone Growth. *PLoS One* 2010;10(2):e0117016.
